# The psychosexual impact of testing positive for high‐risk cervical human papillomavirus (HPV): A systematic review

**DOI:** 10.1002/pon.5198

**Published:** 2019-08-21

**Authors:** Kirsty F. Bennett, Jo Waller, Mairead Ryan, Julia V. Bailey, Laura A.V. Marlow

**Affiliations:** ^1^ Cancer Communication and Screening Group, Department of Behavioural Science and Health University College London London UK; ^2^ e‐Health Unit, Department of Primary Care and Population Health University College London London UK

**Keywords:** cancer, early detection of cancer, oncology, papillomavirus infections, psychological, sexual dysfunctions, systematic review

## Abstract

**Objectives:**

Many countries are implementing human papillomavirus (HPV)‐based cervical screening due to the higher sensitivity of the test compared with cytology. As HPV is sexually transmitted, there may be psychosexual consequences of testing positive for the virus. We aimed to review the literature exploring the psychosexual impact of testing positive for high‐risk cervical HPV.

**Methods:**

MEDLINE, PsycINFO, CINAHL Plus, Web of Science, and EMBASE were searched with no date limits. We also searched the grey literature, reference lists of included articles and carried out forward citation searching. Eligible studies reported at least one psychosexual outcome among HPV‐positive women. Qualitative and quantitative papers were included. We extracted data using a standardised form and carried out a quality assessment for each article. We conducted a narrative synthesis for quantitative studies and a thematic synthesis for qualitative studies.

**Results:**

Twenty‐five articles were included. Quantitative study designs were diverse making it difficult to determine the impact that an HPV positive result would have in the context of routine screening. The qualitative literature suggested that psychosexual concerns cover a broad range of aspects relating to women's current and past relationships, both interpersonal and sexual.

**Conclusions:**

The psychosexual impact of testing positive for high‐risk cervical HPV is unclear. This review highlights the need for further research in the context of HPV‐based cervical screening. As primary HPV testing is introduced more widely, it is important to understand women's responses to testing HPV positive in the cancer screening context to minimise any adverse psychosexual impact.

## 1 BACKGROUND

It is well‐established that virtually all cervical cancers are caused by infection with a high‐risk type of human papillomavirus (hrHPV),^1^, ^2^, ^3^ a very common sexually transmitted infection (STI)^4^ which most sexually active individuals will acquire in their life.^5^ There are many types of HPV, some which do not cause cancer but can cause genital warts or verruca's (low‐risk HPV) and some which can develop into cancer (high‐risk HPV). Fifteen HPV types have been classified as high‐risk.^6^ Although the underlying cause of cervical cancer is infection with hrHPV, infection with hrHPV does not always cause cancer, and most infections resolve spontaneously in less than 2 years.^7^

Until recently, most cervical screening programmes in high‐income countries used cytology to detect cervical abnormalities.^8^ However, HPV primary testing, which will detect the presence of the virus rather than abnormalities, is expected to provide higher sensitivity for identifying high‐grade precancerous disease,^9^, ^10^, ^11^ and several countries have moved, or plan to move, to primary HPV testing. In England, the NHS Cervical Screening Programme is currently rolling this out.

The move to primary HPV testing will change the cervical screening results women receive. In the primary HPV testing pilot in England, approximately 13% of the screened population received an HPV positive result.^12^ Due to the sexually transmitted nature of HPV,^4^ there may be psychosexual consequences of testing positive for the virus. Research suggests that diagnosis with an STI such as genital warts, herpes simplex virus (HSV), or chlamydia can have a negative psychosexual impact. Consequences include reduced sexual desire,^13^, ^14^ reduced sexual satisfaction,^14^, ^15^ and feeling sexually unattractive,^13^ sexually anxious or depressed.^15^ An early qualitative study of HPV testing in cervical screening suggested that similar concerns might apply to women who are told they are HPV positive.^16^

An essential criterion for any screening programme is that the overall benefits should outweigh the harms^16^; therefore, it is important to understand and address any psychosexual consequences of testing positive for HPV, particularly as there will be large numbers of women receiving an HPV positive result. Two previous reviews (published in 2007 and 2009) have explored the psychosexual impact of testing positive for HPV,^17^, ^18^ but the increasing use of HPV testing in cervical screening (e.g., for triage and test of cure) and the current introduction of primary HPV testing have led to significant research activity since these were published. There are also differences between these previous reviews and the current review. One^17^ focused on the economic and quality of life burden of cervical HPV and did not include psychosexual outcomes in the search strategy and the other^18^ had a broad scope and reviewed the psychosexual impact of genital warts and their treatment and HPV‐related genital, oral, and anal precancerous lesions. In advance of the introduction of HPV primary testing in England, we aimed to provide an up‐to‐date systematic review of the qualitative and quantitative literature that has explored psychosexual concerns following an HPV positive test result.

## 2 METHODS

This review was registered with PROSPERO (CRD42018083969) and followed the Preferred Reporting Items for Systematic Review and Meta‐Analysis (PRISMA) guidelines.^19^

### 2.1 Search strategy for identifying papers

The search included terms relating to (a) high‐risk cervical HPV and (b) a psychosexual or disclosure‐related outcome (eg, sexual behaviour, sexual function, and disclosure of HPV status to a partner) and were linked using Boolean operators (see Supporting Information 1 for the search strategy). The search was conducted in MEDLINE, PsycINFO, CINAHL Plus, Web of Science, and EMBASE on 09/01/2019. There were no study design, date, or language limits applied to the initial search, and both qualitative and quantitative papers were included. Additional papers were identified by searching the grey literature using OpenGrey (http://www.opengrey.eu), PsycEXTRA, the reference lists of included articles, and forward citation searching.

### 2.2 Selection process

Studies were included if they mentioned (a) HPV and (b) a psychosexual or disclosure‐related outcome. Reviews, conference abstracts, commentaries, opinion pieces, and editorials were excluded. Studies were also excluded if they were not in English, explicitly focused only on low‐risk HPV or focused on the psychosexual impact of cervical cancer, treatment for cervical cancer, or colposcopy.

Titles were screened by K.B. Two reviewers (K.B. and M.R.) screened the abstracts of the remaining papers (agreement rate = 85%). Where a paper could not be assessed using the abstract, the fulltext was obtained. Disagreements were resolved by discussion.

### 2.3 Data extraction

Using a standardised data extraction form (see Supporting Information 2), one reviewer (K.B.) extracted information from each paper. A second reviewer (M.R.) independently extracted information for 20% of the studies. Extracted data included participant characteristics, study methods, and a summary of psychosexual outcomes. Inconsistencies were resolved through discussion.

### 2.4 Quality assessment

The quality of studies was assessed using modified versions of the National Institute for Health and Care Excellence (NICE) quality appraisal checklists for quantitative and qualitative studies (see Supporting Information 3 and 4). Quality assessment was carried out by one reviewer (K.B.) with a second reviewer (M.R.) independently conducting 20% of assessments. The agreement rate was 80%. Disagreements regarding study quality were resolved by discussion.

### 2.5 Analysis

Quantitative and qualitative findings were analysed separately. For quantitative studies, a narrative synthesis was conducted and the results reported descriptively. We used Popay et al's^20^ framework for narrative synthesis, following three of the suggested elements: (a) a preliminary synthesis of findings was developed, (b) relationships in the data were explored, and (c) the robustness of the synthesis was assessed.

For qualitative studies, we conducted a thematic synthesis, following the three stages outlined by Thomas and Harden^21^: (a) Line‐by‐line coding of text in the results and discussion sections; (b) “descriptive themes” were identified; and (c) “analytic themes” were generated—this involves “going beyond” the content of the studies to generate new interpretive constructs or explanations.

A coding frame was developed and applied to the data (by K.B.). A second reviewer (M.R.) independently coded 20% of these papers, and any inconsistencies were resolved through discussion.

## 3 RESULTS

### 3.1 Search results

**Figure 1:**
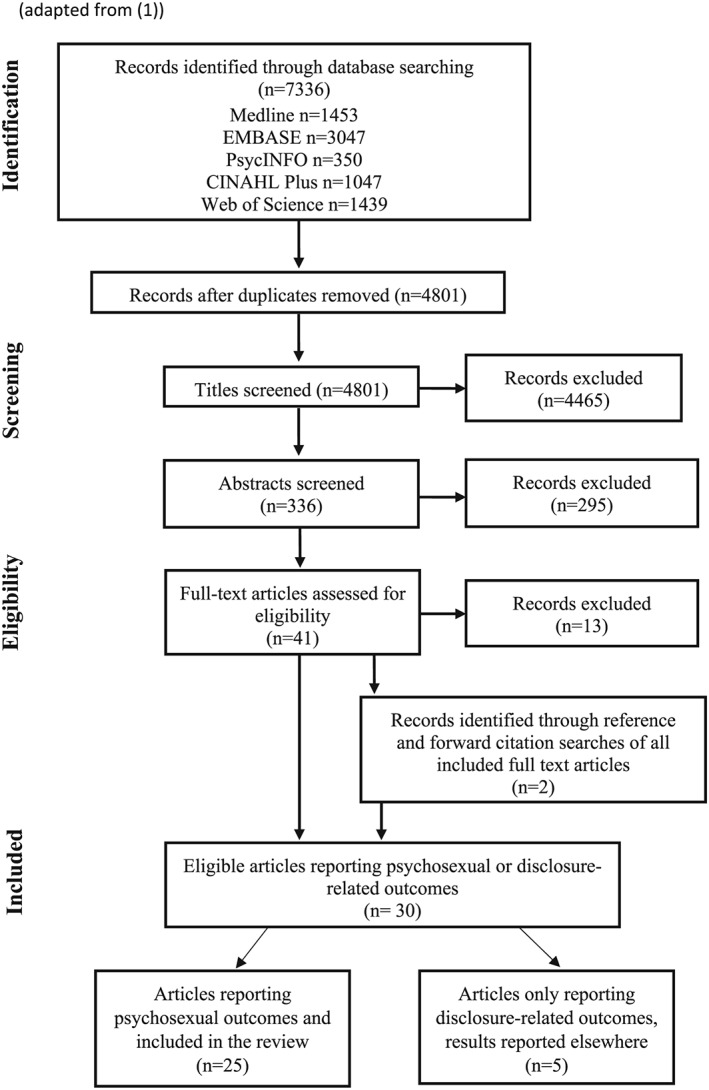
Flow diagram of study selection(adapted from Walboomers et al^1^)

The search yielded 4801 articles after the removal of duplicates. Following exclusions, 40 fulltexts were reviewed. Twelve articles were excluded during the full‐text review, and two were included following backward/forward citation searches, resulting in 30 papers (see Figure 1). Twenty‐five studies measured the psychosexual impact of testing positive for HPV and are included in this analysis.^16^, ^22^, ^23^, ^24^, ^25^, ^26^, ^27^, ^28^, ^29^, ^30^, ^31^, ^32^, ^33^, ^34^, ^35^, ^36^, ^37^, ^38^, ^39^, ^40^, ^41^, ^42^, ^43^, ^44^ The remaining studies described disclosure‐related outcomes only and are not included in the analysis.

Studies were conducted in the United Kingdom (n = 7), United States (n = 5), Taiwan (n = 4), Australia (n = 2), Greece, Hong Kong, Italy, China, Brazil, Sweden, and Belgium (all n = 1) and were published between 1988 and 2018. Studies were quantitative (n = 12; see Table 1) and qualitative (n = 13; see Table 2). All quantitative studies used survey‐based methods,^22^, ^23^, ^24^, ^25^, ^26^, ^27^, ^28^, ^29^, ^30^, ^31^, ^32^, ^33^ and most (n = 8) compared women who were HPV positive (HPV+) with women who were HPV negative (HPV−).^25^, ^26^, ^27^, ^28^, ^29^, ^30^, ^32^, ^33^ Validated measures included the HPV Impact Profile (n = 3), Psychosocial Effects of Abnormal Pap Smears Questionnaire (n = 2), Symptom Checklist of Sexual Function, Sexual Rating Scale, Brief Index of Sexual Functioning of Women, and Psychosocial Adjustment to Illness Scale‐SR (all n = 1). Aspects of psychosexual functioning reported in quantitative studies included sexual satisfaction and pleasure (n = 7), frequency of sex (n = 4), sexual interest, thoughts about sex and sexual arousal (n = 4), and feelings about sexual partners and sexual relationships (n = 4). Some quantitative studies reported an overall psychosexual impact score (n = 6). Most qualitative studies (n = 12) conducted individual interviews.^16^, ^35^, ^36^, ^37^, ^38^, ^39^, ^40^, ^41^, ^43^, ^44^, ^45^

**Table 1:** Characteristics of quantitative studies measuring psychosexual outcomes included in the review

Reference	Country	Age (y)	Psychosexual Outcomes Measured	Number of Participants	Survey Instrument	Time of Data Collection	Study Population	Comparison Groups	Quality Assessment Score Internal Validity/External Validity†
Campion et al (1988)^22^	UK	17‐26	Sexual interest, frequency of sex, sexual arousal, orgasm, negative feelings about sex.	105	Questionnaire	Baseline: 6 mo before attending colposcopy or a genitourinary clinic. Follow‐up: approximately 5‐6 mo later.	Women attending a colposcopy or a genitourinary clinic	1. Abnormal smear test result and cervical intraepithelial neoplasia (CIN), HPV+. 2. Women traced as the regular sexual partner of a man with penile condylomata acuminata: a) HPV+ or HPV+ with CIN. b) No cervical disease. 3. Women referred as the regular sexual partner of a man diagnosed with urethritis who had no evidence of cervical disease.	++/+
Ferenidou et al (2011)^23^	Greece	20‐50+	Sexual interest, frequency of sex, sexual satisfaction, orgasm, impact on relationships (measured by the Symptom Checklist of Sexual Function).	51	Questionnaire	Questionnaire completed after a gynaecological examination, having been diagnosed with HPV at a previous visit.	Women attending a gynaecological clinic.	HPV+ participants only.	+/+
Hsu et al (2018)^24^	Taiwan	20‐61	Effect on sexual relationships (measured by the Psychosocial Effects of Abnormal Pap Smears Questionnaire [baseline] and Psychosocial Adjustment to Illness Scale‐SR [1, 6, and 12 mo follow‐up])	70	Questionnaire	Baseline: at the first follow‐up appointment after HPV diagnosis Follow‐up: 1, 6, and 12 mo following diagnosis.	Women attending a gynaecological clinic	HPV+ participants only.	+/+
Kitchener et al (2007)^25^	UK	20‐64	Sexual satisfaction (measured by the Sexual Rating Scale)	2508	Questionnaire data initially collected in face‐to‐face interviews (n = 106) and subsequently by postal questionnaire	2 wks after receiving screening results.	Women eligible for routine cervical screening in the National Cervical Screening Programme	Revealed arm: 1. HPV−, normal cytology 2. HPV+, normal cytology 3. HPV−. mild/borderline cytology 4. HPV+, mild/borderline cytology Concealed arm: 1. HPV−, normal cytology 2. HPV+, normal cytology 3. HPV−. mild/borderline cytology 4. HPV+, mild/borderline cytology	++/++
Kwan et al (2011)^26^	Hong Kong	36.8 (mean)	Relationship and sexual satisfaction, overall psychosocial burden (which included sexual impact and concerns about infectivity and transmission). (measured by the HPV Impact Profile)	299	Questionnaire	Baseline: after result disclosure. Follow‐up: 6 mo later.	Women attending routine cervical screening	1. ASCUS, HPV+ 2. ASCUS, HPV−	+/+
Maggino et al (2007)^27^	Italy	20‐45	Sexual interest, sexual thoughts, frequency of sex, sexual arousal, sexual satisfaction, sexual pleasure, orgasm (measured by the Brief Index of Sexual Functioning for Women)	72	Questionnaire	Time between HPV diagnosis and questionnaire delivery varied; participants received the questionnaire 0‐6 mo after diagnosis (50%), 6‐12 mo after diagnosis (39%), or 1 + y after diagnosis (11%).	Women attending a gynaecological clinic	1. HPV+ 2. HPV−	+/+
Maissi et al (2005)^28^	UK	Mean age by group: Abnormal, HPV+: 32.7 Abnormal, HPV−: 41.6 Abnormal, HPV not tested: 36.6	Effect on sexual relationships. (measured by the Psychosocial Effects of Abnormal Pap Smears Questionnaire)	1011	Postal questionnaire	Baseline: sent within a week of the research team being informed that an individual's screening test result had been sent. Follow‐up: 6 mo later.	Women undergoing routine cervical screening at one of two centres taking part in the English pilot study of liquid‐based cytology and HPV testing	1. Abnormal cytology, HPV+ 2. Abnormal cytology, HPV− 3. Abnormal cytology, HPV not tested.	++/++
McCaffery et al (2004)^29^	UK	20‐64	Feelings about current, previous, and future sexual partners.	271	Postal questionnaire	One week after receiving screening test results.	Women attending a National Health Service (NHS) well‐woman clinic for routine cervical screening.	1. Normal cytology, HPV+ 2. Normal cytology, HPV− 3. Abnormal/unsatisfactory cytology, HPV+ 4. Abnormal/unsatisfactory cytology, HPV−	++/++
Reed et al (1999)^30^	USA	18‐50	Sexual thoughts, frequency of sex, sexual arousal, sexual satisfaction, negative feelings about relationships.	169	Postal questionnaire	Participants enrolled in a Vaginitis study for at least 6 mo were asked to assess current psychosexual activities and changes in these activities since enrolment, without specific reference to HPV infection.	Women enrolled in the University of Michigan Vaginitis study.	1. HPV+ 2. HPV−	++/+
Wang et al (2010)^31^	Taiwan	18‐65	Sexual impact, concerns about infectivity and transmission (measured by the HPV Impact Profile)	249	Face‐to‐face interviews	Within 3 mo of an HPV‐related diagnosis.	Women recruited from outpatient clinics at three hospitals during routine gynaecological visits.	1. Normal Pap 2. Abnormal Pap 3. CIN 1/2/3 4. Genital warts 5. Abnormal Pap, HPV+	+/++
Wang et al (2011)^32^	China	18‐65	Sexual impact, concerns about infectivity and transmission (measured by the HPV Impact Profile)	2605	Questionnaire completed in the presence of a trained interviewer.	Within 3 mo of an HPV‐related diagnosis.	Women attending routine clinical hospital visits.	1. Normal Pap 2. Abnormal Pap, no HPV test 3. Genital warts 4. Precancer 5. Abnormal Pap, HPV+ 6. Abnormal Pap, HPV−	++/++
Youngkin et al (1998)^33^	USA	17‐29+	Sexual satisfaction (measured by the Self‐Concept and Satisfaction with Intimate Relationships Scale)	58	Questionnaire given during a clinic visit and returned by post.	Baseline: when participants were randomised. Follow‐up: 4 wk after baseline questionnaire.	Women from a university student health service and a family planning clinic.	1. HPV+, self‐help module plus routine counselling (intervention group). 2. HPV+, routine counselling (control group)	++/+

† ++ Indicates that the study was designed or conducted in such a way as to minimise the risk of bias.

+ Indicates that the study was partly designed to minimise bias, may not have addressed all potential sources of bias, or it was not clear from the way the study was reported.

− Indicates that the study had significant sources of bias across all aspects of the study design.

**Table 2:** Characteristics of qualitative studies measuring psychosexual outcomes included in the review

Reference	Country	Age (y)	Number of Participants	Study Design	Study Population	Quality Assessment Score†
Kosenko et al (2012)^44^	USA	19‐56	25	Semi‐structured interviews	Women answering an advertisement posted online (on social media websites and support groups) and in community settings.	++
Jeng et al (2010)^34^	Taiwan	27‐52	20	Semi‐structured interviews	Women attending a gynaecological outpatient clinic at a university‐based hospital.	−
Kosenko et al (2014)^35^	USA	19‐56	25	Semi‐structured interviews	Women answering an advertisement posted online (on social media websites and support groups) and in community settings	++
Lin et al (2011)^36^	Taiwan	27‐56	20	Semi‐structured interviews	Women attending a gynaecological outpatient clinic at a university‐based hospital.	+
McCaffery et al (2006)^16^	UK	20‐64	74	In‐depth interviews	Women taking part in clinical trials of HPV testing or attending colposcopy clinics where HPV testing was used.	++
McCaffery & Irwig (2005)^37^	Australia	Range unknown, 53% were < 35 y; 47% were > 35 y.	19	In‐depth, unstructured interviews	Women attending family planning clinics, general practices and specialist gynaecologist practices.	++
McCurdy et al^38^	USA	21‐45	18	In‐depth interviews	Women attending three private primary care clinics. Women had atypical squamous cells of undetermined significance (ASCUS) or a low‐grade squamous intraepithelial lesion as well as a high‐risk HPV type.	++
Newton & McCabe (2008)^39^	Australia	19‐59	60 (30 with HPV)	Semi‐structured interviews	Men (n = 30) and women (n = 30) responding to an advertisement about the study posted on STI websites, support groups, and online communities.	+
Parente Sa Barreto et al (2016)^40^	Brazil	20‐42	14	Semi‐structured interviews	Women attending a specialised unit supporting sexual and reproductive care. First‐time attenders were excluded from the study.	+
Patel et al (2018)^45^	UK	25‐63	46	Semi‐structured interviews	Women recruited from colposcopy clinics and community settings.	+
Rask et al (2017)^41^	Sweden	29‐53	10	Individual interviews	Women attending a women's health clinic who had been diagnosed with CIN 1/2/3.	++
Waller et al (2007)^43^	UK	21‐64	30	Semistructured interviews	Women participating in the ARTISTIC trial (a randomised trial of HPV testing in primary cervical screening).	++
Verhoeven et al (2010)^42^	Belgium	Not specified	527 email messages (n = 432 from women).	Qualitative analysis of questions asked by visitors to an HPV website.	Individuals who emailed questions about HPV to a website with HPV information.	++

† ++ Indicates that the study was designed or conducted in such a way as to minimise the risk of bias.

+ Indicates that the study was partly designed to minimise bias, may not have addressed all potential sources of bias, or it was not clear from the way the study was reported.

− Indicates that the study had significant sources of bias across all aspects of the study design.

### 3.2 Quality assessment

Most of the quantitative studies were judged to have been designed or conducted in such a way as to minimise the risk of bias and had good internal validity (n = 7). The quality of external validity was mixed. Most qualitative studies were judged to be well conducted (n = 12) (see Tables 1 and 2 for details).

### 3.3 Quantitative studies

#### 3.3.1 Overall psychosexual impact

Six studies reported an overall psychosexual impact score.^24^, ^25^, ^26^, ^28^, ^31^, ^32^ Study designs (including measures used, comparison groups, and point of data collection) were diverse making it challenging to summarise the overall psychosexual impact of testing HPV+.

In a study of women with abnormal cytology in England,^28^ women who were HPV+ had significantly more worries about their sexual health 6 months after receiving their results (compared with women who were HPV− and women not tested for HPV). Two studies (in Taiwan and China) collected data from women who had a range of HPV‐related diagnoses around 3‐months post‐diagnosis.^31^, ^32^ In both studies, women with abnormal cytology who were also HPV+ had similar sexual impact profiles to those with abnormal cytology who were not tested for HPV. Whilst these groups were not directly compared, both groups scored significantly higher than women with normal cytology who were not tested for HPV. In the latter of these studies,^32^ a group of women who were HPV− with abnormal cytology were also included and had similar sexual impact profiles to those who were HPV+, but again, these groups were not directly compared.

Another study^26^ reported an overall psychosocial impact score which included questions on sex, relationship issues, and concerns about transmitting HPV. Psychosocial scores at result notification were worse in women who were HPV+ than women who were HPV− (all women had abnormal cytology), and although scores decreased 6 months later in both groups, they were still significantly worse in women who were HPV+.^26^ However, since this scale assessed a range factors, it is unclear if the between‐group differences were driven by psychosexual or more general concerns.

In a Chinese study of women who were HPV+, many of whom also had abnormal cytology,^24^ psychosexual impact was reported shortly after HPV diagnosis and 1, 6, and 12 months later. At diagnosis, 14% of women had mean subscale scores indicating “significant distress.” At the follow‐up time‐points, psychosexual impact was assessed using a different scale, but all mean scores were low.

In one large, high‐quality study of women tested for HPV in England,^25^ psychosexual functioning was assessed approximately 2 weeks after women received their results. Among women with normal cytology, psychosexual functioning did not differ between those who received an HPV+ or HPV− result. However, among women with abnormal cytology (mild/borderline), psychosexual functioning was better in women who were HPV+ than women who were HPV−.

#### 3.3.2 Sexual satisfaction and pleasure

Seven studies assessed sexual satisfaction or sexual pleasure, with three reporting no impact of testing HPV+.^26^, ^27^, ^30^ In a study of 72 women attending a gynaecological clinic,^27^ there were no significant differences in sexual satisfaction or sexual pleasure/orgasm between women who were HPV+ and women who were HPV− approximately 6 to 12 months post‐diagnosis. In a second study of 155 women with vaginitis,^30^ there were no significant differences in sexual satisfaction between women who were HPV+ and women who were HPV−. A third study of 299 women with abnormal cytology^26^ found no difference in sexual satisfaction at baseline (result notification) or 6 months later between women who were HPV+ and women who were HPV−.

A randomised controlled trial of 58 women who were HPV+ and 40 women who were HSV+ (exploring the effect of counselling and providing information on HPV or HSV) found that, in the control group (who only received counselling), women who were HPV+ had slightly greater satisfaction with intimate relationships than women who were HSV+; however, in the experimental group women with HPV had slightly lower satisfaction with intimate relationships than women with HSV. In this study, the HPV and HSV groups were not statistically directly compared, and the range of potential scores was not reported.

In a descriptive study of 51 women who had recently been informed that they were HPV+,^23^ 22% reported feeling dissatisfied with their sex life, and 22% experienced problems reaching orgasm following HPV diagnosis. In another study of 105 women attending a colposcopy or genitourinary clinic,^22^ frequency of orgasm among women who were HPV+ (with or without cervical intraepithelial neoplasia [CIN]) decreased between baseline (6‐months prior to diagnosis) and follow‐up (6‐months post‐treatment). There was no change in frequency of orgasm among women without HPV.

#### 3.3.3 Frequency of sex

Four studies assessed frequency of sex following an HPV+ result.^22^, ^23^, ^27^, ^30^ In a descriptive study of 51 women who had recently been told they were HPV+,^23^ 41% reported decreased frequency of sex following HPV diagnosis. In a study of 105 women attending a colposcopy or genitourinary clinic,^22^ frequency of sex among women who were HPV+ (with or without CIN) decreased between baseline (6months prior to diagnosis) and follow‐up (6months post‐treatment). There was no change in frequency of sex among women without HPV.

Two studies reported no difference in frequency of sex between women who were HPV+ and women who were HPV−.^27^, ^30^ In a study of 72 women attending a gynaecological clinic,^27^ there were no significant differences in sexual satisfaction between women who were HPV+ and women who were HPV− approximately 6 to 12 months following HPV diagnosis. In a second study of 155 women who had been taking part in a study about vaginitis for at least 6 months,^30^ there were no significant differences between women who were HPV+ and women who were HPV−.

#### 3.3.4 Interest in sex, thoughts about sex, and sexual arousal

Four studies assessed interest in sex, thoughts about sex, and sexual arousal following HPV diagnosis.^22^, ^23^, ^27^, ^30^ In a descriptive study of 51 women who were recently told they were HPV+,^23^ 41% reported decreased sexual desire. In a second study, women who were HPV+ (with or without CIN) who were attending a colposcopy or a genitourinary clinic^22^ reported decreased spontaneous interest in sex and sexual arousal and increased negative feelings towards sexual intercourse between baseline (6months prior to diagnosis) and follow‐up (6months post‐treatment). There was no change in interest in sex among women without HPV. In contrast, among 72 women attending a gynaecological clinic,^27^ there were no significant differences in interest in sex, sexual arousal, or sexual thoughts between women who were HPV+ and women who were HPV− 6 to 12+ months after their visit. In a fourth study of 155 women participating in a study about vaginitis,^30^ there were no differences in sexual arousal or thinking about sex between women who were HPV+ and women who were HPV−.

#### 3.3.5 Feelings about partners and relationships

Four studies assessed feelings about partners and relationships.^23^, ^26^, ^29^, ^30^ In a study of 51 women who had recently been told they were HPV+,^23^ 12% reported feeling their relationship was negatively affected by their result. In a second study of 271 women, conducted in the context of routine cervical screening,^29^ women who were HPV+ (with normal or abnormal cytology) were more likely to report feeling worse about their current, previous, and future sexual partners than women who were HPV− 1 week after receiving their test result.

Two studies found no evidence that an HPV+ result affected feelings about partners or relationships.^26^, ^30^ One study of 299 women with abnormal cytology^26^ reported no differences between women who were HPV+ and women who were HPV− in relationship satisfaction at result notification or 6months later. In a second study of women participating in a study about vaginitis,^30^ there were no significant differences between women who were HPV+ and women who were HPV− in frequency of negative feelings about relationships, or anger at current or previous partner.

### 3.4 Qualitative studies

A thematic synthesis of 13 studies identified three major themes relating to psychosexual impact: (a) source of HPV infection, (b) transmission of HPV, and (c) impact of HPV on sex and relationships. Supporting Information 5 gives a brief description of each theme and provides example quotes.

#### 3.4.1 Source of HPV infection

##### Where did the infection come from?

A common response from women with HPV was to question which partner (current or previous) the infection had come from.^16^, ^35^, ^37^, ^38^, ^42^, ^43^, ^44^, ^45^ Not knowing the source of the infection sometimes led to uncertainty and stress^35^, ^44^ and in severe cases to relationship breakdown^44^ or angry confrontation with a previous partner.^35^

##### Infidelity concerns

Some women expressed concerns that their partner had been unfaithful.^16^, ^34^, ^40^, ^42^, ^43^ Lack of trust was described.^40^ A small number of women were concerned about being accused of infidelity,^38^, ^40^ and there were reports that partners had left due to infidelity concerns,^38^ though this was uncommon.

#### 3.4.2 Transmission of HPV

##### Transmitting HPV to a partner

Concern about passing HPV on to a partner was common.^16^, ^36^, ^37^, ^38^, ^41^, ^42^ Women had questions about the likelihood of infecting their partner^37^ and which sexual practices could lead to infection.^42^ Women wondered what they could do to avoid passing on the infection.^37^ There was uncertainty and a desire for information about the consequences of HPV for male partners.^37^

##### Being re‐infected with HPV

Worry about re‐infection and recurrence was common.^43^ In some cases, this led to concerns about having new partners, because of a fear of being re‐infected.^34^ Some women were worried about infecting their partner and then their partner re‐infecting them, not allowing the virus to be cleared and increasing the risk of cervical cancer.^37^, ^42^

#### 3.4.3 Impact of HPV on sex and relationships

##### Impact of HPV on relationships

Whilst some women were concerned HPV might negatively impact their relationship^36^, ^38^; others reported that it had not. A small number reported that their partners were accepting,^39^ supportive,^38^, ^45^ had shown concern for their wellbeing,^45^ and that they had become closer to their partner following HPV diagnosis.^39^ A small number described their HPV diagnosis having a negative impact on their relationship, feeling that their partner was distant from them,^45^ or that HPV was causing conflict.^36^, ^39^

##### Frequency and interest in sex

Several studies identified a reduced interest in and frequency of sex,^34^, ^36^, ^38^, ^39^, ^42^ with some women reporting that they had stopped having sex.^34^, ^36^, ^39^ Some thought that people with HPV should not have sex,^34^ whilst others were concerned about passing the infection on. There was also concern that having sex would worsen any abnormal cervical cells.^16^

##### Negative sexual self‐image

HPV had a negative impact on some women's sexual self‐image.^16^, ^39^, ^43^, ^46^ The stigma associated with HPV led women to feel “*dirty*,” “*contaminated,*” and unworthy of sexual attention.^16^, ^39^, ^41^ The stigma of having an STI sometimes restricted sexual advances towards others, affected sexual spontaneity, and made women feel they had to alter their sexual activities.^39^

##### Concerns about risks associated with oral sex

The risks associated with oral sex were mentioned by a few women^37^, ^44^ who were concerned about passing HPV on to their partners in this way, with the potential for it to cause oral cancer. This sometimes resulted in abstention from oral sex.

## 4 DISCUSSION

This review synthesises the existing literature on the psychosexual impact of testing positive for high‐risk cervical HPV. The diversity of quantitative study designs and inclusion of study populations with abnormal cytology or other conditions makes it difficult to determine the impact that an HPV+ result would have in the context of routine primary HPV testing; however, some studies suggested that testing HPV+ can have a psychosexual impact. The qualitative literature suggested that psychosexual concerns are raised by some women who test HPV+ and that these concerns cover a broad range of aspects relating to their current and past relationships, both interpersonal and sexual.

Including quantitative and qualitative articles in the review allowed us to highlight the range of psychosexual concerns that women testing HPV+ have. Traditional psychosexual measures used in the quantitative studies assessed specific aspects of sexual behaviour in line with medical classifications of psychosexual disorders (eg, sexual interest and arousal^47^). Conversely, the qualitative literature suggested that the concerns of women with HPV are more about where the infection came from, infectivity, and the impact this can have on relationships. Concerns about infectivity were only assessed by two quantitative studies included in the review, both of which had used qualitative research when developing their questionnaire. Assessing the prevalence of other concerns raised in the qualitative literature is important. Including these aspects in quantitative measures would ensure a more inclusive assessment of the components that influence psychosexual outcomes in women who have HPV.

Previous studies have shown that receiving an abnormal cytology result can have a negative impact on frequency of sex,^22^, ^48^ interest in sex,^22^, ^49^ and satisfaction with sex.^48^ The quantitative studies included in this review that compared HPV+ and HPV− women with abnormal cytology found inconsistent evidence of psychosexual impact.^26^, ^28^, ^31^, ^32^ Our findings both differ and are consistent with previous reviews. One review^17^ found that most studies reported changes in women's sexual relationships following a HPV diagnosis and the other^18^ found no conclusive evidence regarding the psychosexual consequences of an HPV diagnosis.

Comparison groups, measures, and the setting from which participants were recruited differed between studies, and psychosexual outcome data were collected at different time points (from immediately after the test result to more than a year later). The heterogeneity in study design and time from receipt of HPV test results to when data were collected could provide an explanation for the mixed findings, and this makes it difficult to form conclusions about the prevalence and severity of the psychosexual impact of an HPV+ diagnosis. Whilst some studies included in the review did use validated measures, a validated measure specific to HPV testing that assesses aspects of psychosexual and interpersonal relationships (discussed in the qualitative literature) would help to ensure contextually valid items are included and provide a tool that can allow comparisons between studies. Only two papers included in the review measured psychosexual impact longitudinally. Future studies should measure the psychosexual impact of testing HPV+ over time to ascertain if psychosexual impact changes. Knowledge of when psychosexual impact is greatest could help to determine when interventions are most appropriate.

### 4.1 Study limitations

Since the quantitative papers included a range of psychosexual outcomes, it was not possible to conduct a meta‐analysis. Whilst we excluded any articles that explicitly focused on low‐risk types of HPV, some of the papers included in the review did not describe the type of HPV participants had and it is possible that some articles included participants with low‐risk HPV.

### 4.2 Clinical implications

It is important to understand, and minimise, any psychosexual impact of testing HPV+ in the context of primary HPV testing. In line with previous studies (52,53), the qualitative synthesis highlights that women who test HPV+ have a number of questions about HPV such as the source of the infection, whether partners can re‐infect each other and how to prevent the transmission of HPV. Information materials could increase knowledge and address some of these concerns. Additionally, health care professionals carrying out cervical screening could be trained to give brief information during screening to ensure that women understand their results when they receive them. Whilst HPV is classified as an STI, it differs from other STI's as it is normally asymptomatic, does not need treatment, and does not usually cause any long‐term problems. Communicating this information to women is important and may help to reduce psychosexual impact.

## 5 CONCLUSIONS

This review synthesises the literature on the psychosexual impact of testing HPV+. The qualitative studies included in the review provide rich information about the source and nature of psychosexual distress experienced by some women. In particular, women were concerned about transmitting HPV to a partner and where the HPV infection came from. The diversity of quantitative study designs and samples makes it difficult to draw conclusions about the magnitude of psychosexual impact in the context of primary HPV testing. Whilst this review draws together what is currently known, it also highlights the need for further quantitative and qualitative research in the context of primary HPV testing. It is important to understand the psychosexual impact of testing HPV+ in a routine context to minimise undue concern among women, and to avoid compromising future screening re‐attendance.

## ACKNOWLEDGEMENTS

KB is funded by a Medical Research Council (MRC) studentship (grant reference: MR/N013867/1). JW, MR and LM are funded by a Cancer Research UK career development fellowship awarded to JW (grant reference: C7492/A17219).

## CONFLICT OF INTEREST STATEMENT

No conflicts of interest to declare.

## Supporting information

Table S1. Search strategy

Table S2. Data extraction form

Table S3. Quality appraisal checklist—quantitative studies

Table S4. Quality appraisal checklist—qualitative studies

Table S5. A brief description of themes relating to the psychosexual impact of testing positive for high‐risk cervical HPV and the studies associated with them

## References

[1] Walboomers JMM , Jacobs MV , Manos MM , et al. Human papillomavirus is a necessary cause of invasive cervical cancer worldwide. J Pathol. 1999;189(1):12‐19. 10451482 10.1002/(SICI)1096-9896(199909)189:1<12::AID-PATH431>3.0.CO;2-F

[2] Bosch FX , Manos MM , Muñoz N , et al. Prevalence of human papillomavirus in cervical cancer: a worldwide perspective. JNCI: J Natl Cancer Inst. 1995;87(11):796‐802. 7791229 10.1093/jnci/87.11.796

[3] Bosch FX , Lorincz A , Muñoz N , Meijer CJLM , Shah KV . The causal relation between human papillomavirus and cervical cancer. J Clin Pathol. 2002;55(4):244‐265. 11919208 10.1136/jcp.55.4.244 PMC1769629

[4] Satterwhite CL , Torrone E , Meites E , et al. Sexually transmitted infections among US women and men: prevalence and incidence estimates, 2008. Sex Transm Dis. 2013;40(3):187‐193. 23403598 10.1097/OLQ.0b013e318286bb53

[5] World Health Organisation . Human papillomavirus (HPV) and cervical cancer. 2016 Available from: http://www.who.int/mediacentre/factsheets/fs380/en/.

[6] Muñoz N , Bosch FX , de Sanjosé S , et al. Epidemiologic classification of human papillomavirus types associated with cervical cancer. N Engl J Med. 2003;348(6):518‐527. 12571259 10.1056/NEJMoa021641

[7] World Health Organisation . Comprehensive cervical cancer control: a guide to essential practice. 2nd ed. ; 2014. 25642554

[8] Ebell MH , Thai TN , Royalty KJ . Cancer screening recommendations: an international comparison of high income countries. Public Health Rev. 2018;39(1):7. 29507820 10.1186/s40985-018-0080-0 PMC5833039

[9] Ronco G , Dillner J , Elfström KM , et al. Efficacy of HPV‐based screening for prevention of invasive cervical cancer: follow‐up of four European randomised controlled trials. The Lancet. 2014;383(9916):524‐532. 10.1016/S0140-6736(13)62218-7 24192252

[10] Ronco G , Giorgi‐Rossi P , Carozzi F , et al. Efficacy of human papillomavirus testing for the detection of invasive cervical cancers and cervical intraepithelial neoplasia: a randomised controlled trial. Lancet Oncol. 2010;11(3):249‐257. 20089449 10.1016/S1470-2045(09)70360-2

[11] Cuzick J , Clavel C , Petry KU , et al. Overview of the European and North American studies on HPV testing in primary cervical cancer screening. Int J Cancer. 2006;119(5):1095‐1101. 16586444 10.1002/ijc.21955

[12] Rebolj M , Rimmer J , Denton K , et al. Primary cervical screening with high risk human papillomavirus testing: observational study. BMJ. 2019;364:l240. 30728133 10.1136/bmj.l240 PMC6364146

[13] Mortensen GL , Larsen HK . The quality of life of patients with genital warts: a qualitative study. BMC Public Health. 2010;10(1):113. 20205944 10.1186/1471-2458-10-113 PMC2848198

[14] Cai T , Mondaini N , Migno S , et al. Genital Chlamydia trachomatis infection is related to poor sexual quality of life in young sexually active women. J Sex Med. 2011;8(4):1131‐1137. 21269400 10.1111/j.1743-6109.2010.02194.x

[15] Newton DC , McCabe M . Effects of sexually transmitted infection status, relationship status, and disclosure status on sexual self‐concept. J Sex Res. 2008;45(2):187‐192. 18569539 10.1080/00224490802012909

[16] McCaffery K , Waller J , Nazroo J , Wardle J . Social and psychological impact of HPV testing in cervical screening: a qualitative study. Sex Transm Infect. 2006;82(2):169‐174. 16581749 10.1136/sti.2005.016436 PMC2564695

[17] Fleurence RL , Dixon JM , Milanova TF , Beusterien KM . Review of the economic and quality‐of‐life burden of cervical human papillomavirus disease. Am J Obstet Gynecol. 2007;196(3):206‐212. 17346523 10.1016/j.ajog.2007.01.028

[18] Graziottin AS , Serafini A . HPV infection in women: psychosexual impact of genital warts and intraepithelial lesions. J Sex Med. 2009;6(3):633‐645. 19170869 10.1111/j.1743-6109.2008.01151.x

[19] Moher D , Liberati A , Tetzlaff J , Altman DG , The PG . Preferred reporting items for systematic reviews and meta‐analyses: the PRISMA statement. PLoS Med. 2009;6(7):e1000097. 19621072 10.1371/journal.pmed.1000097 PMC2707599

[20] Popay J , Roberts H , Sowden A , et al. Guidance on the conduct of narrative synthesis in systematic reviews: a product from the ESRC Methods Programme. Lancaster Institute of Research: Lancaster; 2006.

[21] Thomas J , Harden A . Methods for the thematic synthesis of qualitative research in systematic reviews. BMC Med Res Methodol. 2008;8(1):45. 18616818 10.1186/1471-2288-8-45 PMC2478656

[22] Campion MJ , Brown JR , McCance DJ , et al. Psychosexual trauma of an abnormal cervical smear. Br J Obstet Gynaecol. 1988;95(2):175‐181. 2831933 10.1111/j.1471-0528.1988.tb06848.x

[23] Ferenidou F , Salakos N , Vaidakis N , et al. The impact of HPV diagnosis on women's sexual and mental health: preliminary findings. Clin Exp Obstet Gynecol. 2012;39(1):79‐82. 22675962

[24] Hsu YY , Wang WM , Fetzer SJ , Cheng YM , Hsu KF . Longitudinal psychosocial adjustment of women to human papillomavirus infection. J Adv Nurs. 2018;74(11):2523‐2532. 29845650 10.1111/jan.13725

[25] Kitchener HC , Fletcher I , Roberts C , Wheeler P , Almonte M , Maguire P . The psychosocial impact of human papillomavirus testing in primary cervical screeninga study within a randomized trial. Int J Gynecol Cancer. 2008;18(4):743‐748. 17944916 10.1111/j.1525-1438.2007.01113.x

[26] Kwan TT , Cheung AN , Lo SS , et al. Psychological burden of testing positive for high‐risk human papillomavirus on women with atypical cervical cytology: a prospective study. Acta Obstet Gynecol Scand. 2011;90(5):445‐451. 21306349 10.1111/j.1600-0412.2011.01092.x

[27] Maggino T , Casadei D , Panontin E , et al. Impact of an HPV diagnosis on the quality of life in young women. Gynecol Oncol. 2007;107(1):S175‐S179. 17825395 10.1016/j.ygyno.2007.07.013

[28] Maissi E , Marteau TM , Hankins M , Moss S , Legood R , Gray A . The psychological impact of human papillomavirus testing in women with borderline or mildly dyskaryotic cervical smear test results: 6‐month follow‐up. Br J Cancer. 2005;92(6):990‐994. 15785734 10.1038/sj.bjc.6602411 PMC2361952

[29] McCaffery K , Waller J , Forrest S , Cadman L , Szarewski A , Wardle J . Testing positive for human papillomavirus in routine cervical screening: examination of psychosocial impact. BJOG—an Int J Obstetrics Gynaecol. 2004;111(12):1437‐1443. 10.1111/j.1471-0528.2004.00279.x 15663132

[30] Reed BD , Mack T IV , Gorenflo DW , Zazove P . The psychosexual impact of human papillomavirus cervical infections. J FAM PRACTICE. 1999;48(2):110‐116. 10037541

[31] Wang KL , Jeng CJ , Yang YC , et al. The psychological impact of illness among women experiencing human papillomavirus‐related illness or screening interventions. J Psychosom Obstet Gynecol. 2010;31(1):16‐23. 10.3109/01674820903564440 20121461

[32] Wang SMS , Shi JF , Kang DJ , Song P , Qiao YL . Impact of human papillomavirusyrelated lesions on quality of life: a multicenter hospital‐based study of women in Mainland China. Int J Gynecol Cancer. 2011;21(1):182‐188. 21330842 10.1097/IGC.0b013e3181ffbed8

[33] Youngkin EQH , Henry JK , Gracely‐Kilgore K . Women with HSV and HPV: a strategy to increase self‐esteem. Clin Excell Nurse Pract. 1999;2(6):370‐375. 12596840

[34] Jeng CJ , Lin H , Wang LR . The effect of HPV infection on a couple's relationship: a qualitative study in Taiwan. Taiwan J Obstet Gynecol. 2010;49(4):407‐412. 21199740 10.1016/S1028-4559(10)60090-3

[35] Kosenko KA , Harvey‐Knowles J , Hurley RJ . The information management processes of women living with HPV. J Health Commun. 2014;19(7):813‐824. 24580554 10.1080/10810730.2013.864728

[36] Lin H , Jeng CJ , Wang LR . Psychological responses of women infected with cervical human papillomavirus: a qualitative study in Taiwan. Taiwan J Obstet Gynecol. 2011;50(2):154‐158. 21791300 10.1016/j.tjog.2011.01.035

[37] McCaffery K , Irwig L . Australian women's needs and preferences for information about human papillomavirus in cervical screening. J Med Screen. 2005;12(3):134‐141. 16156944 10.1258/0969141054855238

[38] McCurdy S , Fernández ME , Arvey S , et al. Hispanic women's concerns about disclosure of their HPV+ status. Hispanic Health Care Int. 2011;9(4):168‐173.

[39] Newton DC , McCabe MP . Sexually transmitted infections: impact on individuals and their relationships. J Health Psychol. 2008;13(7):864‐869. 18809636 10.1177/1359105308095058

[40] Parente Sa Barreto JA , Alexandre MN , Vidal F , et al. Feelings of women with human papilloma virus regarding their infection: an exploratory study. Online Brazilian J Nurs. 2016;15(3):382‐392.

[41] Rask M , Swahnberg K , Lindell G , Oscarsson M . Women's experiences of abnormal Pap smear results – A qualitative study. Sex Reprod Healthc: official journal of the Swedish Association of Midwives. 2017;12:3‐8. 10.1016/j.srhc.2017.01.002 28477928

[42] Verhoeven V , Baay MFD , Baay PE , Lardon F , Van Royen P , Vermorken JB . Everything you always wanted to know about HPV (but could not ask your doctor). Patient Educ Couns. 2010;81(1):101‐105. 20056371 10.1016/j.pec.2009.12.006

[43] Waller J , McCaffery K , Kitchener H , Nazroo J , Wardle J . Women's experiences of repeated HPV testing in the context of cervical cancer screening: a qualitative study. Psychooncology. 2007;16(3):196‐204. 16858719 10.1002/pon.1053

[44] Kosenko KA , Hurley RJ , Harvey JA . Sources of the uncertainty experienced by women with HPV. Qual Health Res. 2012;22(4):534‐545. 22068044 10.1177/1049732311424404

[45] Patel H , Moss EL , Sherman SM . HPV primary cervical screening in England: women's awareness and attitudes. Psychooncology. 2018;27(6):1559‐1564. 29521462 10.1002/pon.4694

[46] Koliopoulos G , Nyaga VN , Santesso N , et al. Cytology versus HPV testing for cervical cancer screening in the general population. Cochrane Database Syst Rev. 2017;8:Cd008587. 28796882 10.1002/14651858.CD008587.pub2 PMC6483676

[47] American Psychiatric Association . Diagnostic and Statistical Manual of Mental Disorders (Fifth Edition). Washington, DC: American Psychiatric Association; 2013.

[48] Drolet M , Brisson M , Maunsell E , et al. The psychosocial impact of an abnormal cervical smear result. Psychooncology. 2012;21(10):1071‐1081. 21695747 10.1002/pon.2003

[49] Wardle J , Pernet A , Stephens D . Psychological consequences of positive results in cervical cancer screening. Psychol Health. 1995;10(3):185‐194.

