# Supplementary material for: The psychosexual impact of testing positive for high‐risk cervical human papillomavirus (HPV): A systematic review
Source: Psychooncology. 2019 Aug 21;28(10):1959–70. doi: 10.1002/pon.5198 (PMC6851776; doi:10.1002/pon.5198)
Supplement: Supplementary file 1 — Table S1. Search strategy [file PON-28-1959-s001.docx]

Supporting Information 5: A brief description of themes relating to the psychosexual impact of testing positive for high-risk cervical HPV and the studies associated with them.

| Theme | Sub-theme | Studies | Explanation | Example quote(s) |
| --- | --- | --- | --- | --- |
| Source of HPV infection | Where did the infection come from? | Kosenko et al. (46)  Kosenko et al. (37)  Lin et al. (38) McCaffery and Irwig (39) McCaffery et al. (18)  McCurdy et al. (40)  Waller at al. (45)  Verhoeven et al. (44) | Women questioned who they had got the infection from. | *'I was thinking how did I get it? How was it transmitted before?...Did I already have the virus with me or did he give me the virus or what's going on?'* (P)(40)^[[1]](#footnote-1)^.  *'The stressful part is I honestly don't know where it came from. That's one thing that would really put me at ease a little bit, if I knew how I got it'* (P)(46). |
|  | Infidelity concerns | Jeng et al. (36)  Lin et al. (38)  McCaffery et al. (18)  McCurdy et al. (40)  Parente Sa Barreto et al. (42)  Waller et al. (45)  Verhoeven et al. (44) | Women wondered whether their partner had been unfaithful | *‘I was angry with my partner, I trusted him blindly and I was disappointed, but he denies cheating on me, however I don’t trust him completely’* (P)(42).  *‘After I found out I have HPV, I don’t trust in my partner as I used to, and now I am suspicious of him all the time…’* (P)(42). |
| Transmission of HPV | Transmitting HPV to a partner | Lin et al. (38)  McCaffery and Irwig (39)  McCaffery et al. (18)  McCurdy et al. (40)  Verhoeven et al. (44)  Rask et al. (43) | Women were concerned about transmitting the infection to their partner. | *'I was absolutely terrified that I would pass on the infection'* (P)(18). |
|  | Being re-infected with HPV | Jeng et al. (36)  McCaffery and Irwig (39)  Waller et al. (45)  Verhoeven et al. (44) | Women with a current partner were concerned that they and their partner would keep re-infecting one another, not allowing the HPV infection to clear. Women not in a relationship were concerned that they may be re-infected by a future partner. | *'…I am not ready to have a boyfriend at present for fear he will give me this kind of infection again'* (P)(36).  *‘I have HPV, and probably my husband will have it too. Won’t we infect each other all the time?’* (P)(44). |
| Impact of HPV on sex and relationships | Impact of HPV on relationships | Kosenko et al. (46) Jeng et al. (36)  Lin et al. (38)  McCurdy et al. (40)  Newton & McCabe (41)  Parente Sa Barreto et al. (42)  Waller et al. (45)  Rask et al. (43) | General comments, positive or negative, about the impact on HPV on relationships. | *‘I found out I had HPV three years into my current relationship. Nothing changed. He still accepts me and respects me regardless of HPV’*(P)(41). |
|  | Frequency and interest in sex | Jeng et al. (36)  Lin et al. (38)  McCurdy et al. (40)  Newton & McCabe (41) Verhoeven et al. (44) | Women reported that their interest in, and frequency of sex, decreased following HPV infection. | *‘No desire for lovemaking’* (P)(36).  *'Sex is no longer in the picture and abstinence is the best way'* (P)(41). |
|  | Negative sexual self-image | McCaffery et al. (18)  Newton and McCabe (41)  Waller et al. (45)  (43)  Rask et al. (43) | Women reported negative feelings about themselves following HPV infection. | *‘I feel like I am a less desirable woman since I have contracted HPV. I feel that most men will reject me and that I am not going to be wanted anymore’* (P)(41). |
|  | Concerns about risks associated with oral sex | Kosenko et al. (46)  McCaffery and Irwig (39) | Women were concerned about passing the infection on during oral sex and the potential for it to lead to oral cancer. | *‘I think it can lead to, if you have oral sex, to mouth cancer, too. I thought I read somewhere or heard that from somebody. So I’m like, God, now I can’t even have oral sex! I don’t have oral sex either way, giving or receiving, because of that’* (P)(47). |

1. (P) denotes a participant comment; (A) denotes an author comment. Number denotes the number of the study in the reference list. [↑](#footnote-ref-1)
