# Supplementary material for: The psychosexual impact of testing positive for high‐risk cervical human papillomavirus (HPV): A systematic review
Source: Psychooncology. 2019 Aug 21;28(10):1959–70. doi: 10.1002/pon.5198 (PMC6851776; doi:10.1002/pon.5198)
Supplement: Supplementary file 2 — Table S2. Data extraction form [file PON-28-1959-s002.docx]

Supporting Information 1
Search Strategy

|  | **MEDLINE, EMBASE, PsycINFO** | **CINAHL Plus** | **Web of Science** |
| --- | --- | --- | --- |
| **HPV** | 1. HPV.mp. 2. "Human Papilloma Virus".mp. 3. "Human Papillomavirus".mp. 4. exp Papillomavirus Infections/ 5. "Cervical intraepithelial neoplasia".mp. 6. Cervical Intraepithelial Neoplasia/ 7. "Genital Warts".mp. 8. Condylomata Acuminata/ 9. "Cervical Dysplasia".mp. 10. Uterine Cervical Dysplasia/ | 1. HPV 2. “Human Papilloma Virus” 3. “Human Papillomavirus” 4. MH “Papillomavirus Infections” 5. “Cervical intraepithelial neoplasia” 6. MH “Cervical Intraepithelial Neoplasia” 7. “Genital Warts” 8. MH “Warts, Veneral” 9. “Cervical Dysplasia” | 1. HPV 2. “Human Papilloma Virus” 3. “Human Papillomavirus” 4. “Cervical Intraepithelial Neoplasia” 5. “Genital Warts” 6. “Cervical Dysplasia” |
| **PSYCHOSEXUAL OUTCOMES** | 1. Psychosexual.mp. 2. Psychosocial.mp. 3. Psych*.mp. 4. “Quality of Life”.mp 5. "Quality of Life"/ 6. Sexual Dysfunctions, Psychological/ 7. “Sex* Impact” 8. Disclos*.mp. 9. Disclosure/ | 1. Psychosexual 2. Psychosocial 3. Psych* 4. “Quality of Life” 5. MH “Quality of Life” 6. (MH “Sexual Dysfunction, Female”) OR (MH “Psychosexual Disorders”) 7. “Sex* Impact” 8. Disclos* | 1. Psychosexual 2. Psychosocial 3. Psych* 4. “Quality of Life” 5. “Sex* Impact” 6. “Sex* Function*” 7. Disclos* |
| **SEARCH COMBINATIONS** | 1 or 2 or 3 or 4 or 5 or 6 or 7 or 8 or 9 or 10  12 or 13 or 14 or 15 or 16 or 17 or 18 or 19 or 20  11 and 21 | 1 or 2 or 3 or 4 or 5 or 6 or 7 or 8 or 9  11 or 12 or 13 or 14 or 15 or 16 or 17 or 18  10 and 19 | 1 or 2 or 3 or 4 or 5 or 6  8 or 9 or 10 or 11 or 12 or 13 |
