# Supplementary material for: The psychosexual impact of testing positive for high‐risk cervical human papillomavirus (HPV): A systematic review
Source: Psychooncology. 2019 Aug 21;28(10):1959–70. doi: 10.1002/pon.5198 (PMC6851776; doi:10.1002/pon.5198)
Supplement: Supplementary file 3 — Table S3. Quality appraisal checklist—quantitative studies [file PON-28-1959-s003.docx]

Supporting Information 2
Data Extraction Form

The psychosexual impact of testing positive for high-risk cervical human papillomavirus - a systematic review.

| ID Number (on Excel spreadsheet) |  |
| --- | --- |
| Date form completed |  |
| Authors |  |
| Title |  |
| Journal |  |
| Year |  |
| Volume |  |
| Issue |  |
| Pages |  |
| **Participants** |  |
| HPV status determined? | YES NO |
| Type of HPV (HR, HR and LR, unsure) |  |
| Number of participants |  |
| Age range of participants |  |
| Gender of participants |  |
| Other relevant sociodemographics |  |
| **Methods** |  |
| Study design |  |
| Aim of study |  |
| Recruitment method |  |
| Recruitment setting |  |
| Outcomes measured |  |
| Method(s) of analysis |  |
| **Results** |  |
| (Psycho)sexual outcomes reported? | YES NO |
| If yes, give summary of results |  |
| Disclosure outcomes reported? | YES NO |
| If yes, give summary of results |  |
| Other notes |  |
