# Supplementary material for: The psychosexual impact of testing positive for high‐risk cervical human papillomavirus (HPV): A systematic review
Source: Psychooncology. 2019 Aug 21;28(10):1959–70. doi: 10.1002/pon.5198 (PMC6851776; doi:10.1002/pon.5198)
Supplement: Supplementary file 4 — Table S4. Quality appraisal checklist—qualitative studies [file PON-28-1959-s004.docx]

Supporting Information 3
Quality Appraisal Checklist – Quantitative Studies

The psychosexual impact of testing positive for high-risk cervical human papillomavirus - a systematic review.

| **ID Number (on Excel spreadsheet)** |  | |
| --- | --- | --- |
| **Date form completed** |  | |
| **Assessed by** |  | |
| **Authors** |  | |
| **Title** |  | |
| **Journal** |  | |
| **Year** |  | |
| **Volume** |  | |
| **Issue** |  | |
| **Pages** |  | |
| **POPULATION** |  | |
| **Is the source population or source area well described?**  *Was the country, setting, location, population demographics etc. adequately described?* | ++  +  -  NR  NA | Comments: |
| **Is the eligible population or area representative of the source population or area?**  *Was the recruitment of individuals, clusters or areas well defined?*  *Was the eligible population representative of the source? Were important groups under-represented?* | ++  +  -  NR  NA | Comments: |
| **Do the selected participants or areas represent the eligible population or area?**  *Was the method of selection of participants from the eligible population well described?*  *What % of selected individuals or clusters agreed to participate? Were there any sources of bias?*  *Were the inclusion or exclusion criteria explicit and appropriate?* | ++  +  -  NR  NA | Comments: |
| **OUTCOMES** |  | |
| **Were the outcome measures reliable?**  *How reliable were outcome measures (e.g. inter- or intra-rater reliability scores)?*  *Was there any indication that measures has been validated (e.g. validated against a gold standard measure or assessed for content validity?* | ++  +  -  NR  NA | Comments: |
| **Were outcomes relevant?**  *Where surrogate outcome measures were used, did they measure what they set out to measure? (e.g. a study to assess impact on physical activity assesses gym membership – a potentially objective outcome measure – but is it a reliable predictor of physical activity?)* | ++  +  -  NR  NA | Comments: |
| **Was follow-up time meaningful?**  Was follow-up long enough to assess long-term benefits or harms?  Was it too long, e.g. participants lost to follow-up? | ++  +  -  NR  NA | Comments: |
| **ANALYSES** |  | |
| **If applicable, were exposure and comparison groups similar at baseline? If not, were these adjusted?**  *Were there any differences between groups in important confounders at baseline?  If so, were these adjusted for in the analyses (e.g. multivariate analyses or stratification). Were there likely to be any residual differences of relevance?* | ++  +  -  NR  NA | Comments: |
| **Was the study sufficiently powered to detect an intervention effect (if one exists)?**  *A power of 0.8 (that is, it is likely to see an effect of a given size if one exists, 80% of the time) is the conventionally accepted standard. Is a power calculation presented? If not, what is the expected effect size? Is the sample size adequate?* | ++  +  -  NR  NA | Comments: |
| **Were the estimates of effect size given or calculable?**  *Were effect estimates (e.g. relative risks, absolute risks) given or possible to calculate?* | ++  +  -  NR  NA | Comments: |
| **Were the analytical methods appropriate?** *Were important differences in follow-up time and likely confounders adjusted for?  If a cluster design, were analyses of sample size (and power), and effect size performed on clusters (and not individuals)? Were subgroup analyses pre-specified?* | ++  +  -  NR  NA | Comments: |
| **Was the precision of intervention effects given or calculable? Were they meaningful?**  *Were confidence intervals or p values for effect estimates given or possible to calculate?  Were CI's wide or were they sufficiently precise to aid decision-making? If precision is lacking, is this because the study is under-powered?* | ++  +  -  NR  NA | Comments: |
| **SUMMARY** |  | |
| **Are the study results internally valid (i.e. unbiased)?**  *How well did the study minimise sources of bias (i.e. adjusting for potential confounders)?  Were there significant flaws in the study design?* | ++  +  -  NR  NA | Comments: |
| **Are the findings generalisable to the source population (i.e. externally valid)?**  *Are there sufficient details given about the study to determine if the findings are generalisable to the source population? Consider: participants, interventions and comparisons, outcomes, resource and policy implications.* | ++  +  -  NR  NA | Comments: |

| **++** | Indicates that for that particular aspect of study design, the study has been designed or conducted in such a way as to minimise the risk of bias. |
| --- | --- |
| **+** | Indicates that either the answer to the checklist question is not clear from the way the study is reported, or that the study may not have addressed all potential sources of bias for that particular aspect of study design. |
| **−** | Should be reserved for those aspects of the study design in which significant sources of bias may persist. |
| **Not reported (NR)** | Should be reserved for those aspects in which the study under review fails to report how they have (or might have) been considered. |
| **Not applicable (NA)** | Should be reserved for those study design aspects that are not applicable given the study design under review (for example, allocation concealment would not be applicable for case control studies). |

In addition, the reviewer is requested to complete in detail the comments section of the quality appraisal form so that the grade awarded for each study aspect is as transparent as possible.

Each study is then awarded an overall study quality grading for internal validity (IV) and a separate one for external validity (EV):

| ++ All or most of the checklist criteria have been fulfilled, where they have not been fulfilled the conclusions are very unlikely to alter.  + Some of the checklist criteria have been fulfilled, where they have not been fulfilled, or not adequately described, the conclusions are unlikely to alter.  − Few or no checklist criteria have been fulfilled and the conclusions are likely or very likely to alter. |
| --- |
