# Supplementary material for: The psychosexual impact of testing positive for high‐risk cervical human papillomavirus (HPV): A systematic review
Source: Psychooncology. 2019 Aug 21;28(10):1959–70. doi: 10.1002/pon.5198 (PMC6851776; doi:10.1002/pon.5198)
Supplement: Supplementary file 5 — Table S5. A brief description of themes relating to the psychosexual impact of testing positive for high‐risk cervical HPV and the studies associated with them [file PON-28-1959-s005.docx]

Supporting Information 4
Quality Appraisal Checklist – Qualitative Studies

The psychosexual impact of testing positive for high-risk cervical human papillomavirus - a systematic review.

| **ID Number (on Excel spreadsheet)** |  | |
| --- | --- | --- |
| **Date form completed** |  | |
| **Assessed by** |  | |
| **Authors** |  | |
| **Title** |  | |
| **Journal** |  | |
| **Year** |  | |
| **Volume** |  | |
| **Issue** |  | |
| **Pages** |  | |
| **THEORETICAL APPROACH** |  | |
| **Is a qualitative approach appropriate?**  *For example:*   - *Does the research question seek to understand processes or structures, or illuminate subjective experiences or meanings?* - *Could a quantitative approach better have addressed the research question?* | Appropriate  Inappropriate  Not sure | Comments: |
| **Is the study clear in what it seeks to do?**  *For example:*   - *Is the purpose of the study discussed – aims/objectives/research question/s?* - *Is there adequate/appropriate reference to the literature?* - *Are underpinning values/assumptions/theory discussed?* | Clear  Unclear  Mixed | Comments: |
| **STUDY DESIGN** |  | |
| **How defensible/rigorous is the research design/methodology?**  *For example:*   - *Is the design appropriate to the research question?* - *Is a rationale given for using a qualitative approach?* - *Are there clear accounts of the rationale/justification for the sampling, data collection and data analysis techniques used?* - *Is the selection of* *cases/sampling strategy theoretically justified?* | Defensible  Indefensible  Not sure | Comments:  No rationale given for using a qualitative approach. |
| **DATA COLLECTION** |  | |
| **How well was the data collection carried out?**  *For example:*   - *Are the data collection methods clearly described?* - *Were the appropriate data collected to address the research question?*   *Was the data collection and record keeping systematic?* | Appropriately  Inappropriately  Not sure/inadequately reported | Comments:  Not sure if data collection or record keeping were systematic? |
| **Is the context clearly described?**  *For example:*   - *Are the characteristics of the participants and settings clearly defined?* - *Were observations made in a sufficient variety of circumstances* - *Was context bias considered* | Clear  Unclear  Not sure | Comments:  Authors didn’t collect data on whether woman had ever tested HPV+. No info on whether women from the community sample had ever been for cervical screening. |
| **Were the methods reliable?**  *For example:*   - *Was data collected by more than 1 method?* - *Is there justification for triangulation, or for not triangulating?* - *Do the methods investigate what they claim to?* | Reliable  Unreliable  Not sure | Comments: |
| **ANALYSIS** |  | |
| **Is the data analysis sufficiently rigorous?**  *For example:*   - *Is the procedure explicit – i.e. is it clear how the data was analysed to arrive at the results?* - *How systematic is the analysis, is the procedure reliable/dependable?* - *Is it clear how the themes and concepts were derived from the data?* | Rigorous  Not rigorous  Not sure/not reported | Comments:  Short section on data analysis. |
| **Is the data 'rich'?**  *For example:*   - *How well are the contexts of the data described?* - *Has the diversity of perspective and content been explored?* - *How well has the detail and depth been demonstrated?* - *Are responses compared and contrasted across groups/sites?* | Rich  Poor  Not sure/not reported | Comments: |
| **Is the analysis reliable?**  *For example:*   - *Did more than 1 researcher theme and code transcripts/data?* - *If so, how were differences resolved?* - *Did participants feedback on the transcripts/data if possible and relevant?* - *Were negative/discrepant results addressed or ignored?* | Reliable  Unreliable  Not sure/not reported | Comments:  Two authors independently reviewed two transcripts.  No info on how differences were resolved. |
| **Are the findings convincing?**  *For example:*   - *Are the findings clearly presented?* - *Are the findings internally coherent?* - *Are extracts from the original data included?* - *Are the data appropriately referenced?* - *Is the reporting clear and coherent?* | Convincing  Not convincing  Not sure | Comments: |
| **Are the findings relevant to the aims of the study?** | Relevant  Irrelevant  Partially relevant | Comments: |
| **Conclusions**  *For example:*   - *How clear are the links between data, interpretation and conclusions?* - *Are the conclusions plausible and coherent?* - *Have alternative explanations been explored and discounted?* - *Does this enhance understanding of the research topic?* - *Are the implications of the research clearly defined?* - *Is there adequate discussion of any limitations encountered?* | Adequate  Inadequate  Not sure | Comments:  Not sure if the paper enhances understanding of the topic…  Implications described.  Limitations section in the discussion. |
| **Ethics** |  | |
| **How clear and coherent is the reporting of ethics?**  *For example:*   - *Have ethical issues been taken into consideration?* - *Are they adequately discussed e.g. do they address consent and anonymity?* - *Have the consequences of the research been considered i.e. raising expectations, changing behaviour?* - *Was the study approved by an ethics committee?* | Appropriate  Inappropriate  Not sure/not reported | Comments:  Ethical approval obtained. |
| **Overall assessment** |  |  |
| **As far as can be ascertained from the paper, how well was the study conducted? (see guidance notes)** | ++  +  − | Comments: |

++ All or most of the checklist criteria have been fulfilled, where they have not been fulfilled the conclusions are very unlikely to alter.

+ Some of the checklist criteria have been fulfilled, where they have not been fulfilled, or not adequately described, the conclusions are unlikely to alter.

– Few or no checklist criteria have been fulfilled and the conclusions are likely or very likely to alter.
